# Merkel Cell Carcinoma: Changing Practice Patterns and Impact on Recurrence-Free and Overall Survival at a Single Institution and Nationally

**DOI:** 10.1245/s10434-021-10727-2

**Published:** 2021-09-07

**Authors:** Andrew Esposito, Daniel Jacobs, Stephan Ariyan, Anjela Galan, Harriet Kluger, James Clune, Sarah Weiss, Thuy Tran, Kelly Olino

**Affiliations:** 1 Division of Surgical Oncology, Department of Surgery, Yale School of Medicine, New Haven, CT USA; 2 Yale School of Medicine, New Haven, CT USA; 3 Division of Plastic Surgery, Department of Surgery, Yale School of Medicine, New Haven, CT USA; 4 Departments of Dermatology and Pathology, Yale School of Medicine, New Haven, CT USA; 5 Division of Medical Oncology, Department of Medicine, Yale School of Medicine, New Haven, CT USA

## Abstract

**Background:**

Merkel cell carcinoma (MCC) is an aggressive neuroendocrine carcinoma of the skin. Our report describes the evolution of management and characteristics associated with recurrence, disease-specific survival (DSS) and overall survival (OS) in the treatment of MCC.

**Methods:**

A single institution retrospective review of MCC and SEER data to determine factors associated with RFS, DSS, and OS using a multivariable Cox regression on inverse-probability weighted cohorts.

**Results:**

One hundred fifty-nine patients were identified with a median age of 75. Of these, 96% were Caucasian and 60% male. Fifty-eight out of 159 (36%) of all patients were deceased with 21/58 (36%) dead from MCC with a median follow-up of 3.1 years. Institutionally, trends over time demonstrated an increased use of immunotherapy with a concomitant decrease in chemotherapy and decreased use of radiotherapy alone. Institutionally and nationally, there has been increased surgical nodal staging. Institutionally, factors associated with shorter DSS included advanced age, active cigarette smoker (*p* = 0.002), cT2 disease (*p* = 0.007), and MCC with unknown primary (*p* < 0.001). Institutionally, factors associated with shorter OS included ages ≥ 75 years (*p* < 0.001), an immunocompromised state (*p* < 0.001), truncal primary site (*p* = 0.002), and cT2 disease (HR 9.59, *p* < 0.001).

**Conclusion:**

Changing practice patterns in MCC management have been driven by the adoption of immunotherapy. Our study highlights that competing risks of mortality in MCC patients likely prevents OS from being an accurate surrogate outcome measure to understand factors associated with DSS.

In 1972, Toker first described cutaneous trabecular carcinoma (TC),^1^ later reclassified as Merkel cell carcinoma (MCC).^2^ Although the pathogenesis continues to be ill-defined, two likely pathways, one attributed to UV associated mutagenesis and the other related to the Merkel cell polyomavirus (MCPV) appear to be causitive.^3,4^ MCC, which almost exclusively affects patients who are Caucasian, elderly, immunosuppressed, or those with extensive UV exposure,^5^ is associated with poor overall survival (OS) ranging from 63 to 81% for stage I to 11–15% for stage IV disease^6–8^ and can have upwards of 50% recurrence.^9^ While MCC is still a rare disease, its incidence continues to rise, and the United States has seen a 95% increase in the incidence from 0.5 cases per 100,000 in 2000 to 0.66 cases per 100,000 in 2016, with a predicted continued increase to 5130 cases/year by 2030.^3,10^

Historically, treatment for MCC has been variable and included a combination of surgical resection of the primary with or without sentinel lymph node evaluation, radiation of the primary tumor and/or the associated lymph node basin, and chemotherapy, resulting in generally poor durable responses.^11^ Recent clinical trials have demonstrated the efficacy of immunotherapy, while further defining the role of nodal staging and radiotherapy has led to improvements in treatment response.^11–15^ Current treatment guidelines recommend wide local excision with sentinel lymph node biopsy and consideration of adjuvant radiation therapy for all N0M0 MCC.^16^ However, there is considerable debate concerning what factors may affect recurrence-free survival (RFS), disease-specific survival (DSS) and OS of MCC.

While large databases, such as the National Cancer Database (NCDB) or Surveillance, Epidemiology and End Results (SEER) program of the National Cancer Institute, allow for the study of thousands of patients with MCC, they are unable to provide details concerning disease-specific or recurrence-free survival. Previous database driven studies have used overall survival as a surrogate for disease-specific outcomes. However, given the predilection of this disease to occur in older and immunosuppressed patient populations, we sought to determine whether such an approach was appropriate, given a likely competing risk of mortality in this population. Additionally, we aimed to both define the changing practice patterns over time for MCC and to determine characteristics associated with recurrence-free, disease-specific, and overall survival through a retrospective review of a single, high-volume institution and the SEER database.

## Materials and Methods

### Software

SAS 9.4 (SAS Institute Inc., Cary, NC, USA) and Joinpoint Regression Program 4.8.0.1—April 2020 (Statistical Methodology and Applications Branch, Surveillance Research Program, National Cancer Institute) were utilized.

### Patient Selection and Variable Definitions

This was a single institution retrospective review of medical records with patients identified using ICD-9 and ICD-10 codes for MCC diagnosed between 2002 and 2020. In all, 174 patients were identified as having biopsy proven MCC. Fifteen were excluded due to incomplete clinicopathologic data, leaving 159 patients evaluable for analysis. Patient demographics, medical histories, diagnoses, and treatments for MCC were recorded. All staging was based on 8^th^ Edition of American Committee on Cancer (AJCC).^17^

To contextualize changes in management at our institution, we looked at national trends for these parameters using the Surveillance, Epidemiology, and End Results (SEER) Program database. The SEER database was queried for new MCC (histology code 8247) cases diagnosed between 2004 and 2016 for therapy trend analysis, and between 1975 and 2016 for survival analysis. Analysis was performed on the first primary tumor case listed for each patient.

Rates for surgeries performed is accurately documented within the SEER database. However, there remains ambiguity in coding for specific procedures. We documented procedures of biopsy followed by gross excision, Mohs micrographic surgery, wide local excision, and amputations as receipt of surgery. Patients who were coded as having excisional biopsies or local ablations were grouped with patients having received no surgery, as these were deemed likely to not be therapeutic in nature. Patients with unknown surgery to the primary site or LN drainage bed were not included in the proportion analysis. Analysis of surgical management of the primary tumor and lymph node drainage bed was performed on patients with known surgical procedures to the respective sites.

### Statistical Methods

Age-adjusted therapy utilization rates were calculated. Trends in rates were analyzed through Joinpoint regression, and proportions were fitted using simple linear regression. Proportions were calculated from software reported rate ratios. Ninety-five percent confidence intervals (CI) are reported.

For institutional data, RFS, DSS, and OS were assessed using multivariable Cox regressions. For SEER data, DSS and OS were assessed using bivariate Cox regressions. To adjust for the potential selection bias associated with surgical candidates theoretically being “healthier” than non-surgical candidates, inverse-probability weighting (IPW) was used in our survival analyses. Cox regression models were performed using backward elimination with *p*_out_ > 0.05. DSS analysis using the institutional and SEER datasets was performed using competing event Fine and Gray models to account for competing causes of mortality. Patients with an unknown cause of death were not censored from institutional and SEER datasets, and unknown cause of death was treated as a competing cause of mortality.

Overall survival over time was analyzed in the SEER database comparing all-cause mortality in 1975–1986 with the periods 1987–1996, 1997–2006, and 2007–2016 using a univariate Cox regression stratified by patient age (≤ 64, 65–74, 75–84, and ≥ 85 years). Trends in DSS was not assessed given differences in rates reporting patient cause of death over time, which may have reflected differences in reporting as opposed to true differences in survival outcomes.

## Results

### Institutional Patient and Oncologic Information

We identified 159 patients at our institution treated for MCC between 2002 and 2020. Consistent with previous data, 152/159 (96%) of patients were Caucasian, 97/159 (61%) were male, with a median age of 75 (Table 1). At the time of most recent follow-up 58/159 (36%) of all patients were deceased, with 36% (21/58) dead from MCC disease with a median follow-up for all patients of 3.1 years. Twenty-eight of 159 (18%) patients were found to have in-transit disease (ITD): 19 (68%) presented with ITD and 9 (32%) developed ITD during the follow-up period. At the time of presentation, 85/159 (53%) had clinical stage I disease, 34/159 (21%) were clinical stage II, 22/159 (14%) were clinical stage III, 5/159 (3%) were clinical stage IV, and 13/159 (8%) were unknown. After pathologic staging 75/159 (47%) were stage I, 30/159 (19%) were stage II, 42/159 (26%) were stage III, 5/159 (3%) were stage IV, and 7/159 (4%) were unknown.

**Table 1:** Patient demographics and disease characteristics of institutional cohort (*n* = 159)

Median age at diagnosis, years (range)	75 (41–98)
Median follow up, years (range)	3.1 (0–25)
Sex, *n* (%)	
Male	97 (61%)
Female	62 (39%)
Race, *n* (%)	
White	152 (96%)
Other	7 (5%)
Smoking status, *n* (%)	
Never	73 (46%)
Current	16 (10%)
Former	70 (44%)
Lymphoma, *n* (%)	
Yes	8 (5%)
No	150 (94%)
Unknown	1 (1%)
Clinical status, *n* (%)	
Alive	101 (64%)
Dead	58 (36%)
Cause of death (*n* = 56), *n* (%)	
MCC	21 (36%)
Comorbidities	19 (33%)
Other cancer	6 (10%)
Unknown	13 (22%)
Location of primary tumor, *n* (%)	
Head and neck	67 (42%)
Extremity and trunk	80 (50%)
Unknown	12 (8%)
Recurrence, *n* (%)	
Yes	45 (28%)
No	113 (71%)
Unknown	1 (1%)
In-transit disease, *n* (%)	
Yes	28 (18%)
No	131 (82%)
Initial clinical stage, *n* (%)	
Stage I	85 (53%)
Stage II	34 (21%)
Stage III	22 (14%)
Stage IV	5 (3%)
Unknown	13 (8%)
Initial pathologic stage, *n* (%)	
Stage I	75 (47%)
Stage IIa	25 (16%)
Stage IIb	5 (3%)
Stage IIIa	31 (19%)
Stage IIIb	11 (7%)
Stave IV	5 (3%)
Unknown	7 (4%)
Final pathologic stage, *n* (%)	
Stage I	59 (37%)
Stage IIa	16 (10%)
Stage IIb	5 (3%)
Stage IIIa	26 (16%)
Stage IIIb	19 (12%)
Stave IV	28 (18%)
Unknown	3 (2%)

Initial treatments included surgery for both the primary and nodal basin, radiation, immunotherapy, and chemotherapy (Appendix A). Initial surgery was performed on 153/159 (96%) patients. Surgical interventions included: wide local excision (WLE) alone in 56/153 (37%) patients, Mohs in 2/153 (1%) patients, WLE and sentinel lymph node biopsy (SLNB) in 72/153 (47%) patients, WLE and lymph node dissection (LND) in 12/153 (8%) patients, parotidectomy with SLNB in 1/153 (1%) patients, parotidectomy with LND in 2/153 (1%) patients, LND alone in 2/153 (1%) patients, and 6/153 (4%) an unknown surgery. Overall, radiation therapy to the primary or nodal basin was performed in 89/159 (57%) patients, chemotherapy was utilized in 22/159 (14%) patients, and immunotherapy was utilized in 27 (17%) patients.

### Institutional Treatment Trends

**Fig. 1:**
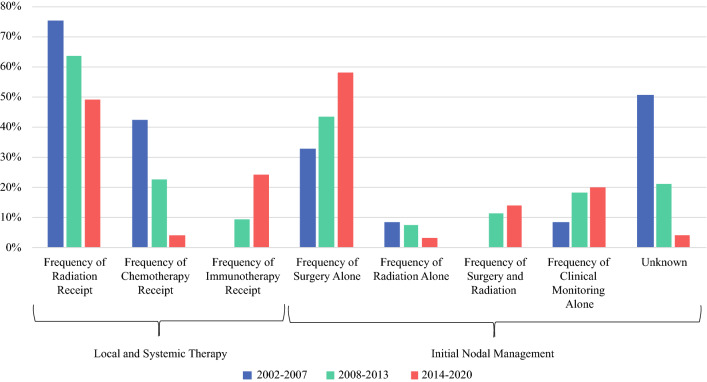
Trends in treatment regimens for Merkel cell carcinoma over time of institutional cohort. The total number of patients treated in each time period was 13 for 2002–2007, 56 for 2008–2013, and 91 for 2014–2020

The frequency of use of these treatment modalities and initial management of lymph node basins changed over time (Fig. 1). The frequency of radiation alone to the lymph node basin dropped from 8 (1/12) in 2002–2008 to 3% (3/91) in 2015–2020. The rate of radiation and surgery together increased over the same time periods from 0 to 14% (13/91). The frequency of surgery as the sole treatment increased from 33 (4/12) to 58% (53/91). In patients with non-distant metastatic (M0) disease, we found that age ≥ 85 years (OR 0.05; 95% CI 0.01, 0.17, *p* < 0.001), cN+ disease (OR 0.14; CI 0.05, 0.45, *p* < 0.001), and ITD at time of diagnosis (OR 0.23; CI 0.07, 0.85, *p* = 0.027) were significantly associated with failure to receive at least WLE of the primary tumor and surgical staging of the lymph nodes (Table 2).

**Table 2:** Factors associated with receipt of guideline-compliant surgical primary tumor excision with lymph node evaluation

Variable	Odds ratio (95% CI)	*p* value
Age (years)		
≤74	Reference	
75–79	0.31 (0.08, 1.17)	0.084
80–84	0.89 (0.12, 1.27)	0.116
≥ 85	0.05 (0.01, 0.17)	< 0.001*
cN		
N0	Reference	
N+	0.14 (0.05, 0.45)	< 0.001*
In-transit disease at diagnosis		
No	Reference	
Yes	0.23 (0.07, 0.85)	0.027^a^

^a^Factors demonstrating significance

Between 3 consecutive 5-year periods the use of radiation therapy dropped from 75 (9/12) to 49% (45/91) of institutional patients. The frequency of chemotherapy decreased from 42 (5/12) to 4% (4/91) of institutional patients, and with the development of newer immunotherapeutics, there was an increase in the frequency of immunotherapy from 0 (0/12) to 24% (22/91) of patients (Fig. 1).

Since the initiation of immunotherapy, 27/91 (30%) of patients received immunotherapy with anti-PD-L1/PD-1 antibodies with 25/27 (93%) initially treated with pembrolizumab (anti-PD-1), 1/27 (3.7%) with nivolumab, and 1 treated with avelumab (PD-L1). Nine out of 27 (36%) of these patients recurred, requiring additional treatment.

### National Treatment Trends

**Fig. 2:**
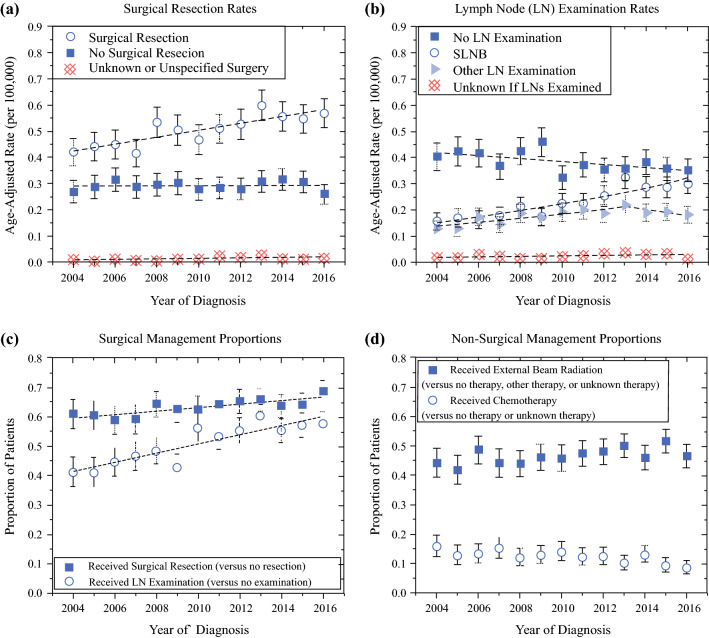
Age-adjusted rates of **a** surgical resection and **b** LN examination. Proportion of patients receiving **c** surgical and **d** non-surgical management. Proportions calculated **c** amongst those with known therapy versus **d** with known or unknown therapy. All data from SEER database. *Error bars* represent 95% confidence intervals

Query of the SEER database revealed 6766 patients with complete data available to allow for the analysis of trends in therapy utilization, whereas a total of 9551 patients were available for survival analysis. When comparing trends in surgical approaches from our experience to those nationally, utilizing patient data from the SEER database, we found that there has been an increase in the utilization of surgery and lymph node examination during the same time period (Fig. 2). The rate of surgical resection increased from 0.422 to 0.578 per 100,000 persons from 2004 to 2016, which corresponded to an average annual percentage change (AAPC) of 2.7% per year (95% CI 1.6, 3.8%) (Fig. 2A). The rate of no surgical resection or unknown/unspecified surgery remained unchanged with AAPCs of 0.3% (CI − 0.9, 1.5%) and 6.3% (CI − 2.1, 15.5%) per year, respectively. In patients with known surgery, surgical resection increased from 61.1% of patients in 2004 to 68.4% of patients in 2016 (trend *p* = 0.002) (Fig. 2B).

Utilization of SLNB increased from 0.156 to 0.300 per 100,000 persons from 2004 to 2016, which corresponded to an average annual percentage change (AAPC) of 6.4% per year (CI 4.6, 8.2%), Fig. 2B. Utilization of other LN examination procedures increased at a rate of 5.2% (CI 2.3, 8.1%) per year until 2013 (CI 2006, 2014) before plateauing at an unchanged rate. The rate of no LN examination or unknown/unspecified LN examination remained unchanged with AAPCs of − 1.2% (CI −2.6, 0.2%) and 4.8% (CI − 0.7, 10.6%) per year, respectively. In patients with known LN examination type, LN examination increased from 41.4% of patients in 2004 to 56.9% of patients in 2016 (trend *p* < 0.001) (Fig. 2C).

Nationally, receipt of radiation was stable between 2004 and 2016 from 44.2 to 46.9%, respectively, while there may have been a decline in utilization of chemotherapy from at least 16.0–8.7% of patients over the same time, respectively (Fig. 2D).

### Survival Analysis

After IPW, for institutional patients with M0 disease, the factors associated with lower RFS were advanced age (≥ 85 years vs. ≤ 64 years, HR 3.32, *p* = 0.045), clinical T2 (HR 9.59, *p* < 0.001), and ITD at diagnosis (HR 2.95, *p* = 0.014) (Table 3). Factors associated with worse DSS included advanced age, being a current cigarette smoker (HR 14.68, *p* = 0.002), cT2 disease (HR 6.37, *p* = 0.007), and MCC with unknown primary (HR 21.79, *p* < 0.001) (Table 4). There were no factors significantly associated with improved RFS or DSS. Factors associated with worse OS included all ages ≥ 75 years (*p* < 0.001), an immunocompromised state (HR 10.62, *p* < 0.001), trunk as the primary site (HR 7.11, *p* = 0.002), and cT2 disease (HR 9.59, *p* < 0.001) (Table 5). Factors associated with improved OS included female sex (HR 0.35, *p* = 0.006), ITD at time of diagnosis (HR 0.27, *p* = 0.032) and treatment with surgery and lymph node evaluation (HR 0.44, *p* = 0.019) (Table 5).

**Table 3:** Factors associated with recurrence-free survival of the institutional cohort

Variable	Hazard ratio (95% CI)	*p* value	*n*
Age (years)			
≤ 74	Reference		73
75–79	1.82 (0.64, 5.16)	0.263	19
80–84	1.83 (0.79, 4.27)	0.160	27
≥ 85	3.32 (1.03, 10.71)	0.045*	22
cT			
T1	Reference		93
T2	2.66 (1.19, 5.93)	0.017*	30
T3–T4	4.80 (1.65, 13.94)	0.004*	10
Unknown	0.05 (0.00, 1.86)	0.106	10
cN			
N0	Reference		118
N+	1.40 (0.54, 3.61)	0.487*	23
In-transit disease at diagnosis			
No	Reference		126
Yes	2.95 (1.24, 7.01)	0.014*	17
Receipt of surgery with lymph node evaluation			
No	Reference		47
Yes	1.48 (0.73, 3.00)	0.277	96

*Factors demonstrating significance

**Table 4:** Factors associated with disease-specific survival of the institutional cohort

Variable	Hazard ratio (95% CI)	*p *value	*n*
Age (years)			
≤74	Reference		73
75–79	6.09 (1.00, 37.03)	0.050*	19
80–84	3.21 (0.73, 14.05)	0.122	27
≥ 85	5.02 (1.38, 18.30)	0.015*	22
Smoking status			
Never/unknown	Reference		66
Current	14.68 (2.82, 79.26)	0.002*	12
Former	2.08 (0.89, 7.41)	0.257	65
cT			
T1	Reference		93
T2	6.37 (1.65, 24.62)	0.007*	30
T3–T4	3.12 (0.50, 19.67)	0.226	10
Unknown	21.79 (4.30, 110.50)	< 0.001*	10
cN			
N0	Reference		118
N+	0.97 (0.23, 4.08)	0.966	23
In-transit disease at diagnosis			
No	Reference		126
Yes	0.91 (0.19, 4.40)	0.911	17
Receipt of surgery with lymph node evaluation			
No	Reference		47
Yes	2.35 (0.89, 6.18)	0.084	96

*Factors demonstrating significance

**Table 5:** Factors associated with overall survival of the institutional cohort

Variable	Hazard ratio (95% CI)	*p *value	*n*
Age (years)			
≤ 74	Reference		73
75–79	7.39 (1.76, 31.04)	0.006*	19
80–84	10.78 (2.91, 39.95)	< 0.001*	27
≥ 85	35.08 (8.31, 148.04)	< 0.001*	22
Sex			
Male	Reference		88
Female	0.35 (0.17, 0.74)	0.006*	55
Immunocompromised state			
No	Reference		135
Yes	10.62 (2.71, 41.64)	< 0.001*	8
Primary site			
Head	Reference		61
Extremity	0.58 (0.26, 1.25)	0.163	66
Trunk	7.11 (2.11, 23.94)	0.002*	7
Unknown	1.46 (0.30, 7.13)	0.639	9
cT			
T1	Reference		93
T2	9.59 (3.26, 28.23)	< 0.001*	30
T3–T4	0.33 (0.03, 3.67)	0.364	10
Unknown	3.48 (0.30, 40.15)	0.318	10
cN			
N0	Reference		118
N+	0.55 (0.09, 3.49)	0.526	23
In-transit disease at diagnosis			
No	Reference		126
Yes	0.27 (0.08, 0.90)	0.032*	17
Receipt of surgery with lymph node evaluation			
No	Reference		47
Yes	0.44 (0.22, 0.87)	0.019*	96

*Factors demonstrating significance

Discrepancies in characteristics associated with RFS, DSS, and OS in the institutional dataset were further evaluated using the SEER database. Analysis of SEER data demonstrated that competing-cause mortality is accentuated with increasing age and increasing time from diagnosis. In patients ≤ 64 years of age, DSS and OS closely mirror each other until approximately 1 year of follow-up, with approximately 83.1% of overall mortality attributable to MCC disease, (Appendices B and C). However, in patients ≥ 85 years, DSS and OS diverge almost immediately, with only 26.6% of overall mortality attributable to MCC disease at 1 year. Mortality associated with MCC remains nearly unchanged after approximately 3 years, and thereafter mortality is almost exclusively a result of death from competing causes, Appendix B. Compared with 1975–1986, OS has not changed over time (HR 0.98, *p* = 0.92 in 1987–1996; HR 0.95, *p* = 0.74 in 1997–2006; and HR 0.86, *p* = 0.34 in 2007–2016).

## Discussion

When considering practice patterns, our institutional experience demonstrated changing trends that were consistent with those seen nationally when compared with the SEER database. At our institution, chemotherapy utilization has declined over the past 2 decades from 42 to 4% whereas the rate of use of immunotherapy has increased from 0 to 24%. This is to be expected given that chemotherapy has not improved survival or reduced the rate of distant metastasis or recurrence.^16,18^ Our analysis also demonstrated a significant increase in the use of immunotherapy to treat MCC. In 2016, two key clinical trials, the KEYNOTE-017 and JAVELIN demonstrated the effectiveness of systemic immunotherapy in treating metastatic MCC, leading to FDA approval.^11–15,19,20^ Current studies including the STAMP trial are underway to better define the use of these treatments in the adjuvant setting (ClinicalTrials.gov NCT03712605). Our institutional data reflects a relatively rapid incorporation of an effective therapy in clinical practice, specifically for patients with in-transit or metastatic disease.

We found that patients who were ≥ 85 years and who had clinically positive lymph nodes were managed in a manner which differed from NCCN guidelines pertaining to surgical excision of the primary tumor and evaluation of the lymph node basin via sentinel lymph node biopsy or lymph node dissection. It is not surprising that older age is associated with deviation from the guidelines and is consistent with findings in patients with breast cancer^21^ and melanoma.^22^ This is likely attributable to patient comorbidities^23^ which may be appropriate in an older population. We and other groups have shown that following national guidelines for staging and management has been associated with improved OS.^24^ It is critically important that major centers who treat MCC prospectively record why patients are not receiving guideline compliant therapy.

We then turned to our analysis of RFS, DSS, and OS. We failed to find any factors that were associated with improved RFS. The factors associated with lower RFS included age ≥ 85 years, cT2 and cT3 or cT4 disease, lymph node positive disease and ITD at time of diagnosis. Interestingly, the receipt of surgery with lymph node evaluation was not associated with improved RFS which contrasts with previously published work.^25,26^ This may be due to the small sample size. While it is appropriate to utilize OS as a surrogate for DSS survival in certain cancers, such as colon cancer,^27^ our analysis indicates that this is less informative in MCC. The median age of our cohort was 75 years, which is consistent with the literature. This age group has multiple competing risks^28^ for mortality outside of their MCC diagnosis. Our institutional data analysis demonstrated that older age and cT2 disease were both associated with worse DSS and OS. In contrast, current smoker status was associated with worse DSS, while an immunocompromised state and truncal tumor primary location were only associated with worse OS. When there is little consistency between the effects each factor has on DSS and OS, this points to the need for caution when utilizing OS alone as an outcome measure. Based upon our analysis, the competing risk for mortality in this patient population leads to this divergence of factors that are associated with DSS and OS.

This idea is further supported through analysis of the SEER database which demonstrated that as age increases, competing-cause mortality accentuates differences in DSS and OS with age and time. This, combined with the inconsistencies we found in factors associated with DSS and OS, demonstrate that OS may be a poor proxy for therapeutic efficacy in retrospective analyses. Indeed, factors associated with OS from large database studies can, and should, be used to pose hypotheses for carefully performed institutional analyses—such as done in this study. However, we demonstrate that without investigation into how such factors associate with RFS or DSS, the real-world application of findings from such OS analyses will remain limited.

This study represents a large academic center’s experience with 159 patients compared with national trends in care seen in the SEER database. There are several limitations of this paper. The data collected is from a single, tertiary academic hospital in the northeast of the United States. This may limit the applicability of the findings. Additionally, this was also a retrospective review of medical records, leading to a risk of information and selection bias. Further study is required of MCC; specifically, institutional studies utilizing disease-specific outcomes, given the rarity of this diagnosis. With the notable rise in the use of immunotherapy, further characterizations to look for predictive biomarkers to response will also be an important avenue of continued research. Further study is also required to elucidate how the etiology, whether from UV radiation or MCPV, affects RFS, DSS, and OS. Currently, our institution does not test for MCPV. Thus, we have no data on the effect of MCPV seropositivity on recurrence-free, overall, and disease-specific survival. Finally, we recommend a stronger emphasis on reporting DSS when discussing MCC given the competing risks of the population affected by MCC, making OS a poor indicator of disease-specific outcomes.

The analysis of national treatment trends must be interpreted with caution. Rates of treatment with radiation therapy and chemotherapy may not be accurately coded, especially in the earlier years. Patients with unknown radiation or chemotherapy information were grouped with patients who did not receive such therapies into a “no/unknown” category by the SEER program. Consequently, rates of receipt likely represent the minimum rate for such therapies, since the positive predictive value for receipt of therapy, with a coding of “yes,” is high. We, therefore, cannot definitively conclude whether rates of radiation therapy or chemotherapy, or their combinations with surgery, have changed. However, we believe that rates and proportions are suggestive for national treatment trends when evaluated within the larger scope of the manuscript. Finally, it was not possible to specifically evaluate the effect of immunotherapy on RFS, OS, and DSS given overall small numbers and relatively short follow-up. However, re-evaluation of this subset of patients to understand the effect of immunotherapy will be critical in the future. Furthermore, while we were able to evaluate trends in OS over time with the SEER dataset, DSS was not assessed given variable rates of cause of death reporting. Such variability did not permit the possibility of interpreting results with any degree of confidence.

## Conclusion

This study demonstrates that the practice patterns in the management of MCC have changed over time both at an institutional and at a national level. These changes appear to match emerging data concerning optimal treatment regimens for MCC, given the significant increase in the utilization of immunotherapy. Further, this study demonstrates that there is a divergence of factors associated with DSS and OS. This indicates that reliance on OS may have limitations in studying MCC outcomes.

## Figures and Tables

**Fig. 3:**
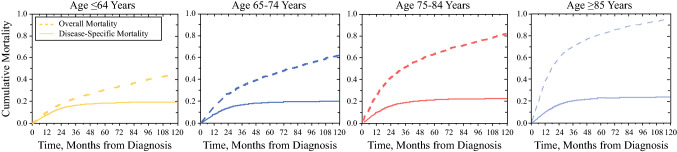
Comparison of overall and disease-specific mortality with increasing age using the SEER database

**Fig. 4:**
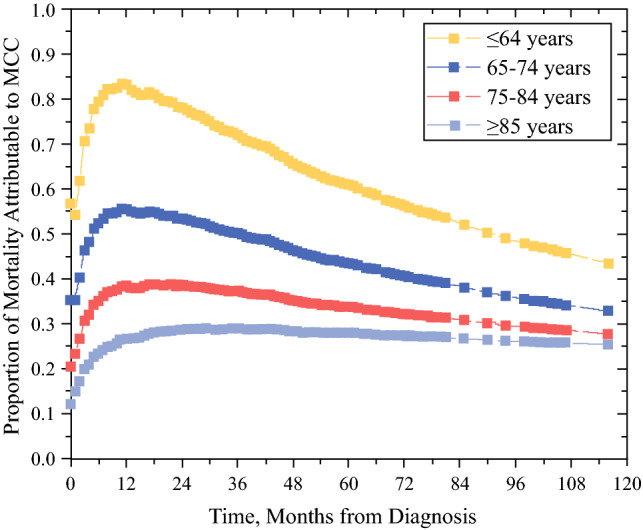
Proportion of mortality attributable to death secondary to MCC over time of SEER database

## Appendix A

See Table 6.

**Table 6:** Treatment characteristics of the institutional cohort

Surgical resection (*n* = 159), *n* (%)	
Yes	153 (96%)
No	6 (4%)
Initial surgical resection targets (*n* = 153), *n* (%)	
Local alone	60 (38%)
Local + regional	87 (57%)
Regional alone	2 (1%)
None	4 (3%)
Unknown	6 (4%)
Radiation receipt (*n* = 159), *n* (%)	
Yes	89 (56%)
No	64 (40%)
Unknown	6 (4%)
Initial radiation target (*n* = 89), *n* (%)	
Primary	42 (47%)
Nodal basin	3 (3%)
Primary and nodal basin	24 (27%)
Metastatic disease	3 (3%)
Unknown	17 (27%)
Chemotherapy receipt (*n* = 159), *n* (%)	
Yes	22 (14%)
No	135 (85%)
Unknown	2 (1%)
Immunotherapy (*n* = 159), *n* (%)	
Yes	27 (17%)
No	127 (80%)
Unknown	5 (3%)

## Appendix B

See Fig. 3.

## Appendix C

See Fig. 4.
